# Intestinal Anti-Inflammatory Activity of Terpenes in Experimental Models (2010–2020): A Review

**DOI:** 10.3390/molecules25225430

**Published:** 2020-11-20

**Authors:** Maria Elaine Araruna, Catarina Serafim, Edvaldo Alves Júnior, Clelia Hiruma-Lima, Margareth Diniz, Leônia Batista

**Affiliations:** 1 Postgraduate Program in Natural Products and Bioactive Synthetic, Health Sciences Center, Federal University of Paraiba, João Pessoa 58051-900, PB, Brazil; elaine.araruna@gmail.com (M.E.A.); catarinaalvesdelima@gmail.com (C.S.); edvaldojunioralves@gmail.com (E.A.J.); margareth@reitoria.ufpb.br (M.D.); 2 Department of Structural and Functional Biology (Physiology), Institute of Biosciences, São Paulo State University, Botucatu 18618-970, SP, Brazil; clelia.hiruma@unesp.br; 3 Department of Pharmacy, Health Sciences Center, Federal University of Paraíba, João Pessoa 58051-900, PB, Brazil

**Keywords:** terpenes, intestinal anti-inflammatory, ulcerative colitis, cytokines, natural products

## Abstract

Inflammatory bowel diseases (IBDs) refer to a group of disorders characterized by inflammation in the mucosa of the gastrointestinal tract, which mainly comprises Crohn’s disease (CD) and ulcerative colitis (UC). IBDs are characterized by inflammation of the intestinal mucosa, are highly debilitating, and are without a definitive cure. Their pathogenesis has not yet been fully elucidated; however, it is assumed that genetic, immunological, and environmental factors are involved. People affected by IBDs have relapses, and therapeutic regimens are not always able to keep symptoms in remission over the long term. Natural products emerge as an alternative for the development of new drugs; bioactive compounds are promising in the treatment of several disorders, among them those that affect the gastrointestinal tract, due to their wide structural diversity and biological activities. This review compiles 12 terpenes with intestinal anti-inflammatory activity evaluated in animal models and in vitro studies. The therapeutic approach to IBDs using terpenes acts basically to prevent oxidative stress, combat dysbiosis, restore intestinal permeability, and improve the inflammation process in different signaling pathways.

## 1. Introduction

Inflammatory bowel diseases (IBDs) refer to a group of disorders characterized by inflammation in the mucosa of the gastrointestinal tract, which mainly comprises Crohn’s disease (CD) and ulcerative colitis (UC) and which are highly debilitating and without definitive cure [1,2,3].

The incidence and prevalence of IBDs vary considerably in different geographic regions [4]. The most massive numbers of cases are concentrated in Europe, North America, and Oceania; in recent years, there has been a growing expansion in Asia, the Middle East, and South America [5,6]. They similarly affect men and women. They are typically diagnosed in individuals between 18 and 35 years of age, although the rate of pediatric diagnosis has increased in recent years [7,8].

On the one hand, CD is characterized by irregular transmural inflammation that can involve the entire gastrointestinal tract. It is generally associated with complications, such as strictures, abscesses, and fistulas. UC, on the other hand, is characterized by inflammation of the mucosa limited to the colon, starting in the rectal region and spreading continuously [9,10].

The etiology of IBDs has not yet been fully clarified. Exposure to environmental factors, genetic susceptibility, and imbalance of the intestinal microbiota play an essential role in the occurrence and progression of these diseases, as well as the uncontrolled response of the immune system against normal enteric microflora [11,12]. The clinical symptoms frequently presented by these diseases are diarrhea, weight loss, nausea, and abdominal pain that affect the quality of life of people who are affected by them [13,14].

Current pharmacotherapy includes the use of corticosteroids, immunosuppressants, 5-aminosalicylates, and biological therapies to reduce the inflammatory process through the immune system [15,16]. However, the therapeutic approach to IBDs may require action on the three main pathophysiological components—dysbiosis, intestinal permeability, and inflammation. It is believed that, in the future, therapies will be highly individualized, based on specific diagnoses, and identify which of these components is the dysfunction [10,15].

Given the complexity of the etiology, new methods for assessing biological consequences in response to these exposures pose a major challenge. The development of better animal models is necessary to study the mechanisms by which environmental exposures can impact remission, disease outbreaks, complications, and response to treatment [17].

Natural products, especially medicinal plants, represent a critical approach to the discovery and development of new medicines [18]. The bioactive compounds derived from medicinal plants appear as a new therapeutic alternative for the treatment of several disorders, among them those that affect the gastrointestinal tract; these products have produced promising results and a decrease in adverse effects [19,20].

The multiple biological activities of plants are due to the diversity of secondary metabolites that bind to macromolecules in the organism [21]. These are grouped according to their synthesis pathway into classes, such as alkaloids, flavonoids, phenylpropanoids, terpenes, among others [20,22].

Terpenes are the most abundant group of secondary plant metabolites; they are the main constituents of essential oils [21]. These metabolites are biosynthesized from the mevalonate (classical) and the methylerythritol phosphate (alternative) pathways. Their basic structure is made up of isoprene units (C_5_H_8_), from isopentenyl pyrophosphate (IPP) and dimethylallyl pyrophosphate (DMAPP). From the arrangement of isoprene units, the classes of these constituents are defined as follows: monoterpenes (C_10_), sesquiterpenes (C_15_), diterpenes (C_20_), triterpenes (C_30_), and tetraterpenes (C4_0_) [23,24].

Several preclinical studies show evidence that terpenes have pharmacological activities such as anti-inflammatory [25], antioxidant [26], antibacterial [27], gastroprotective, and gastric healing [28].

Recently, some reviews have addressed the anti-inflammatory activity of terpenes [29,30], as well as the intestinal anti-inflammatory activity of natural products [31]. The present review aims to compile studies from the last few years, which evaluated the anti-inflammatory activity of terpenes in experimental models of IBDs and mechanisms of action involved.

## 2. Results and Discussion

### 2.1. (+)-Borneol

**Figure 1:**
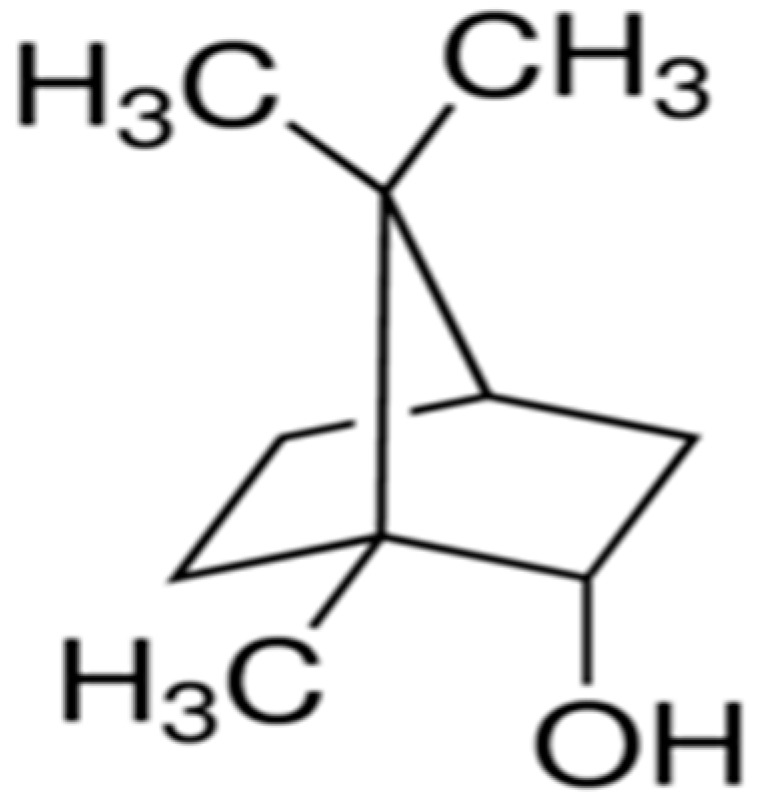
(+)-Borneol.

(+)-Borneol (endo-(1*R*)-1,7,7-trimethylbicyclo[2.2.1]heptan-2-ol) (Figure 1) is a bicyclic monoterpene extracted from essential oils of medicinal plants, such as *Blumea balsamifera* (L.) D.C. of the family Asteraceae or *Cinnamomum camphora* (L.) Presl., widely used in traditional Chinese medicine [32]. Studies have shown pharmacological activities of Borneol as antinociception [33], anti-inflammatory [34], vasorelaxant [35] and neuroprotective, regulating the blood–brain barrier permeability [32].

In a study carried out by Zhang et al. (2017), the authors examined the activity of (+)-borneol and the association with edaravone (EDA) in the model of colitis induced by dextran sulfate sodium (DSS). EDS (3-methyl-1-phenyl-2-pyrazolin-5-one) is a free radical scavenger that shows promising activities in the prevention of neuroinflammation, liver damage, and antioxidants [36]. The study evaluated whether the association could improve the effectiveness of EDA against colitis. Acute colitis was induced by adding 2.5% DSS in drinking water for seven days. The treatments were carried out with EDA (3, 6 or 12 mg/kg), with (+)-borneol (3 mg/kg), or with a preparation composed of EDA and (+)-borneol with a mass ratio of 4:1, respectively (3.75, 7.5 or 15 mg/kg). All treatments were administered intraperitoneally (i.p.) for another ten days after induction [37]. The DSS-induced colitis model has advantages over several other chemically induced experimental models due to its quickness, simplicity, reproducibility, and controllability [38].

DSS is a chemical compound with anticoagulant properties that induce epithelial damage and intestinal inflammation. This results in the rupture of the lining of the intestinal epithelial monolayer, leading to the entry of luminous bacteria and associated antigens in the mucosa and allowing the spread of the pro-inflammatory intestinal content in the underlying tissue [38].

The association of EDA and (+)-borneol at doses of 7.5 and 15 mg/kg significantly reduced the disease activity index (DAI) with reduced body weight loss and colon length in a dose-dependent manner. In contrast, EDA or (+)-borneol alone had moderate effects. Moreover, the immunofluorescence method revealed that the association of EDA and (+)-borneol at doses of 7.5 and 15 mg/kg drastically reduced the levels of inflammatory cytokines (IL-1β, IL-6, and TNF-α) and increased the levels of the anti-inflammatory cytokine associated with M2 macrophages (IL-10) when compared to EDA or (+)-borneol administered alone [37].

Macrophages are innate immune system cells, which play an important role in maintaining intestinal homeostasis. Depending on environmental stimuli, they are generally polarized into two functionally opposed forms, i.e., classically activated macrophages (M1) and alternatively activated macrophages (M2) [39,40]. M1 macrophages induced by IFN-γ, lipopolysaccharide (LPS), TNF-α, and granulocyte-macrophage colony-stimulating factor (GM-CSF) can trigger Th1 and Th17 responses producing high levels of inflammatory cytokines, including TNF-α, IL- 6, IL-1β, IL-12, and IL-23 [41]. In contrast, the M2 macrophages polarized by IL-4, IL-13, and macrophage colony-stimulating factor (M-CSF) participate in the T_h_2 response, exhibiting an anti-inflammatory profile through the positive regulation of IL-10 expression, arginase 1 (Arg-1), and CD206 antigen [40,41].

Studies show that STAT3 is one of the leading transcription factors for polarizing macrophages concerning the M2 phenotype [42,43]. Phosphorylation of STAT3 via the JAK2 signaling pathway promotes the translocation of the STAT3 nucleus and activates the expression of anti-inflammatory factors related to M2 macrophages, such as IL-10 and Arg-1 [43,44].

Association of EDA and (+)-borneol could promote the phosphorylation and translocation of the STAT3 nucleus in vitro, in comparison with the control treatment, with the ability to polarize M2 macrophages and the activation of STAT3 superior to EDA or (+)-borneol isolates [37].

### 2.2. β-Carotene

**Figure 2:**
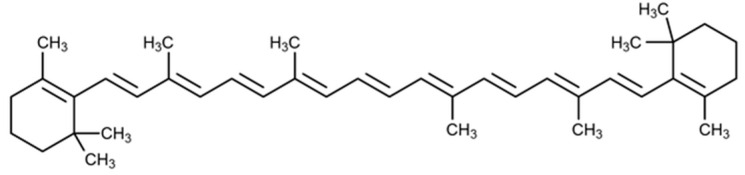
β-Carotene.

β-Carotene (1,3,3-trimethyl-2-[(1*E*,3*E*,5*E*,7*E*,*E*,11*E*,13*E*,1*E*,17*E*)-3,7,12,16-tetramethyl-18-(2,6,6-trimethylcyclohexen-1-yl) octadeca-1,3,5,7,9,11,13,15,17-nonaenyl] cyclohexene) (Figure 2) is a tetraterpene carotenoid, an organic pigment synthesized universally by all photoautotrophs, including macroalgae and plants [45]. It is often found in the human diet metabolically converted to vitamin A [46]. It is widely known for its relevant physiological function as an effective antioxidant [45], inhibits lipid peroxidation, and does not induce genotoxicity [46].

Vitamin A, also known as retinol, is absorbed in the intestine. In its oxidized form, retinoic acid plays an important role in mucosal immunity, immune tolerance, gene expression, differentiation, and function of various immune cells [47,48]. Retinoic acid produced by intestinal cells, thymic stromal lymphopoietin, and TGF-β promotes the development of regulatory dendritic cells, producing IL-10 that stimulates anti-inflammatory responses [49]. The negative regulation of retinoic acid was observed in IBDs [50].

Oral administration in doses of 5, 10 or 20 mg/kg was performed over 28 days of the experiment. UC was induced in mice using 3% *w*/*v* DSS in drinking water during two cycles, namely a cycle composed of seven days of water treated with DSS and followed by one of fourteen days of regular drinking water (representing the period of remission of the disease), and from the 22nd to the 28th day again DSS 3% *w*/*v* [50].

Trivedi and Jena (2015) demonstrated that β-carotene treatment improved the severity of UC by modulating several molecular targets, such as nuclear factor kappa B (NF-κB), cyclooxygenase-2 (COX-2), IL-17, signal transducer and transcription activator 3, factor 2 related to nuclear erythroid 2, metalloproteinase-9 matrix and connective tissue growth factor. The NF-κB acts in the regulation of the inflammatory response, and its translocation from the cytoplasm to the nucleus influences the expression of pro-inflammatory cytokines. The prevention of the nuclear translocation NF-κB can, therefore, act as a potential therapeutic target [51]. IL-17 has a pro-inflammatory activity, which induces cytokine production by increasing the T_h_1 response as well as the expression of chemokines and adhesion molecules by epithelial and endothelial cells [52].

### 2.3. Carvacrol

**Figure 3:**
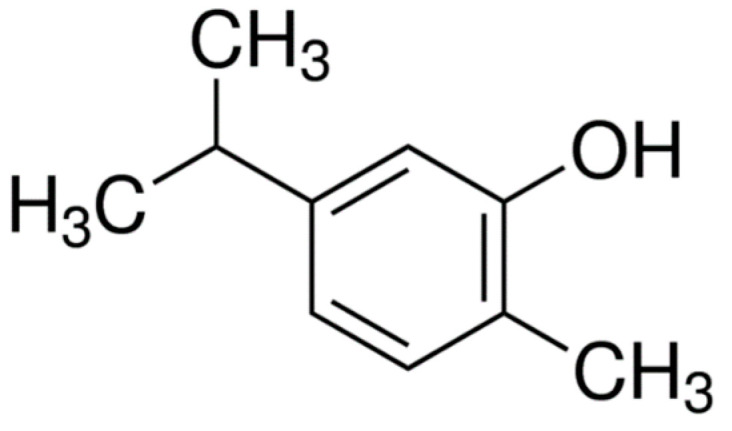
Carvacrol.

Carvacrol (5-isopropyl-2-methylphenol) (Figure 3) is a phenolic monoterpene that has described pharmacological activities, including antioxidant, antibacterial [26], anti-inflammatory [25], cardioprotective [53] antinociceptive, and gastroprotective [54].

To assess the intestinal anti-inflammatory activity of carvacrol, rats were subjected to intrarectal administration of acetic acid (5%) to induce colitis. Pretreatment with carvacrol in doses (25, 50 or 100 mg/kg, p.o.) was performed every 12 h for three days before induction [55].

The intrarectal administration of diluted acetic acid provides an alternative method to create a chemical lesion in the mucosal epithelium that induces a transient phenotype that mimics UC. It generates diffuse colitis related to the dose of acetic acid with histopathological characteristics, such as ulcerative lesions in the distal colon or abnormality in intestinal crypts that extends to the lamina propria [13,56].

Pretreatment with all doses of carvacrol reduced abdominal hyperalgesia, colon myeloperoxidase (MPO) activity, lipid peroxidation, and levels of TNF-α and IL-1β. The authors observed a reduction in macroscopic and microscopic damage (*p* < 0.05) at doses of 50 or 100 mg/kg and an increase in sulfhydryl groups (100 mg/kg) [55]. Studies demonstrated a significant infiltration of neutrophils by humans with UC, with a consequent increase in MPO activity [57,58]. The treatment with carvacrol induced a significant increase in catalase (CAT), superoxide dismutase (SOD), and glutathione peroxidase (GPx) activities. The antioxidant system is a set of molecules and enzymes that react with reactive oxygen species (ROS) and inactivate them to prevent oxidative stress [59]. ROS includes reactive ions and oxygen peroxides that cause damage, in high concentrations, to biomolecules such as DNA, RNA, proteins and lipids, which can lead to homeostasis imbalance [60,61].

The body has an antioxidant defense system that can be enzymatic and non-enzymatic. The enzyme defense system is composed of proteases that form the first line of defense against the superoxide anion and hydrogen peroxide, such as CAT, SOD, GPx, and glutathione S transferase (GST) [59,62].

These findings indicate that the administration of carvacrol acted by reducing inflammatory, nociceptive, and oxidative damage in the model studied [55].

### 2.4. Ganoderic Acid C1

**Figure 4:**
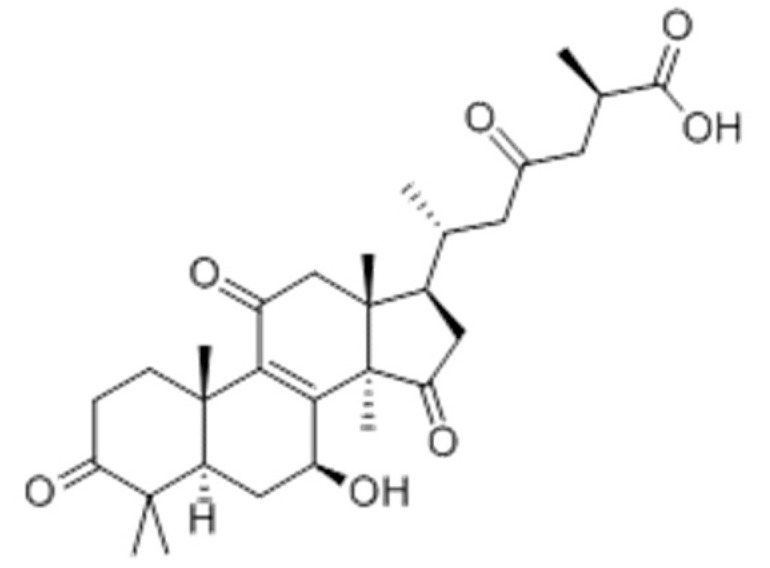
Ganoderic acid C1.

Ganoderic acid C1 (6-(7-hydroxy-4,4,10,13,14-pentamethyl-3,11,15-trioxo-1,2,5,6,7,12,16,17-octahydrocyclopenta[a]phenanthren-17-yl)-2-methyl-4-oxoheptanoic acid) is a triterpenoid isolated from *Ganoderma lucidum* (*G. lucidum*) (Figure 4).

In an in vitro study by Liu et al. (2012), ganoderic acid C1 inhibited the production of TNF-α from a macrophage cell line, through a reduction of NF-κB signaling. Activation of this pathway is involved in inflammatory processes prevalent in neutrophils such as asthma [63]; this condition is also found in CD [64].

Neurath (2014) suggests that the deregulation of intestinal CD4^+^ T-cell subgroups leads to an abnormal immune response to bacterial bacteria in genetically predisposed individuals, as T_h_1 and Th17 cells increase the production of effector cells and generate an imbalance with regulatory T cells. Thus, a CD pathogen includes the positive activation of multiple cytokines, including TNF-α, IFN-γ, IL-1, IL-2, IL-6, IL-12 and IL-17 [3].

In the study by Liu et al. (2015), ganoderic acid C1 (20 or 40 μg/mL) reduced the production of TNF-α by macrophages and blood mononuclear cells from individuals with CD. It decreased the production of IFN-γ and IL-17A in cell biopsies of inflamed colon. Additionally, it inhibited the production of TNF-α and other pro-inflammatory cytokines from mononuclear cells in the blood and inflamed colon mucosa of individuals with CD. These effects were attributed to negative NF-κB signaling. These results justify a clinical investigation for the treatment of CD [64].

### 2.5. Geraniol

**Figure 5:**
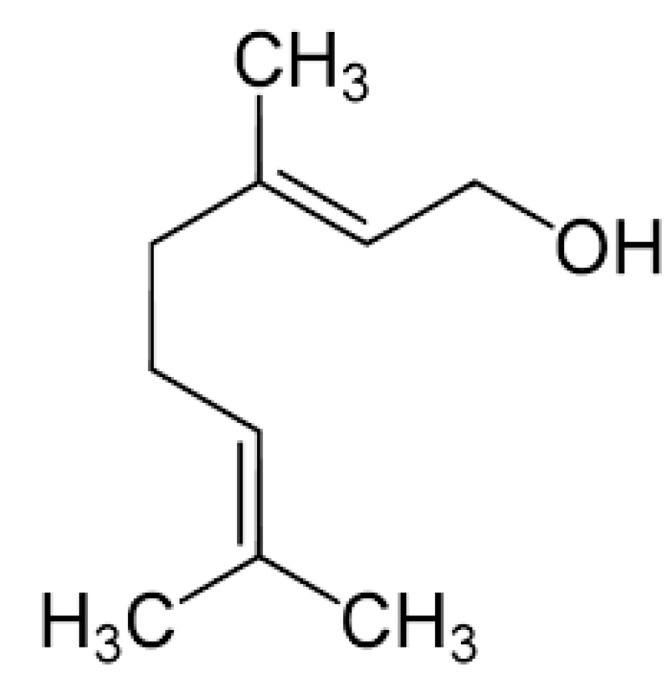
Geraniol.

Geraniol (trans-3,7-dimethyl-2,6-octadien-1-ol) (Figure 5) is an acyclic isoprenoid monoterpene isolated from the essential oils of aromatic plants, including *Cinnamomum tenuipilum*, *Valeriana officinalis*, and several other aromatic plants [65]. It has several pharmacological effects, antioxidant and anti-inflammatory properties [66], gastroprotective activity, and gastric healing [28].

In the study by Soubh et al. (2015), rats were treated with the standard drug sulfasalazine (500 mg/kg p.o.), geraniol (250 mg/kg p.o.), or a combination of geraniol with the standard drug, for 11 days. Trinitrobenzene sulphonic acid (TNBS) was instilled on the eighth day, shortly before administration of treatment.

In this induction model, the TNBS hapten penetrates the intestinal wall, resulting in the haptenization of proteins derived from the colon or microbiota. Subsequently, the generation of specific TCD4^+^ cells and antibodies is observed, making them immunogenic and triggering innate and adaptive immune responses in the host. Administration of TNBS leads to the development of a cell-mediated immune response that reflects a Th1 and Th17 phenotype of inflammation [67,68].

Geraniol significantly reduced the clinical signs of colitis (weight loss, colon edema, ulcerative area, colon/spleen mass indexes), preserved the total antioxidant capacity, and decreased the high levels of nitric oxide (NO) and lipid peroxide. TNBS induced apoptosis and inflammatory cell infiltration, whereas geraniol reduced these effects, decreasing the levels of caspase-3, intercellular adhesion molecule-1, and MPO activity [69].

The anti-inflammatory effect of geraniol was related to inhibition of the colon contents of prostaglandin E2 (PGE2) and IL-1β. In assessing the pathways involved in anticolitic activity, geraniol inhibited the expression of glycogen synthase kinase (GSK)-3β, β-catenin, protein kinase activated by mitogen p38 (p38MAPK), and NF-κB [69]. The anti-inflammatory activity of geraniol can be mediated by inhibiting the NF-κB signaling pathway and the MAPK cascade (ERK, SAP/JNK, and p38MAPK), promoting a reduction in pro-inflammatory cytokines. Furthermore, geraniol upregulated the γ receptor activated by peroxisome proliferator (PPARγ), these effects were comparable to those of sulfasalazine, the standard drug. The effects mediated by the combination of sulfasalazine with geraniol, surpassed any isolated treatment [69].

The role of geraniol as an anti-inflammatory agent in the model with DSS-induced colitis in mice was investigated in another study. Geraniol was administered orally in daily doses of 30 or 120 mg/kg, starting six days before treatment with DSS and ending the day after its removal. Additionally, geraniol (120 mg/kg) was also administered intrarectally, in the enema pharmaceutical form, during the acute phase of colitis to assess its action locally [70].

The results show that geraniol administered orally or as an enema is a powerful antimicrobial agent capable of preventing dysbiosis associated with colitis and reducing the systemic inflammatory profile of colitic animals [70]. Dysbiosis, seen in the imbalance of the intestinal microbiota, as well as the uncontrolled response of the immune system against normal enteric microflora play an essential role in the occurrence and progression of IBDs [71].

It was previously shown that COX-2 mRNA increases significantly in the intestinal wall of mice treated with DSS [72]. COX-2 plays a crucial role in intestinal inflammation, one of the main targets of pharmacological therapy for IBDs. The administration of geraniol greatly improved the clinical signs of colitis. It significantly reduced the expression of COX-2 in the colon and, in the intestinal wall, geraniol could be a powerful substance for the treatment of intestinal inflammation and dysbiosis [70].

### 2.6. d-Limonene

**Figure 6:**
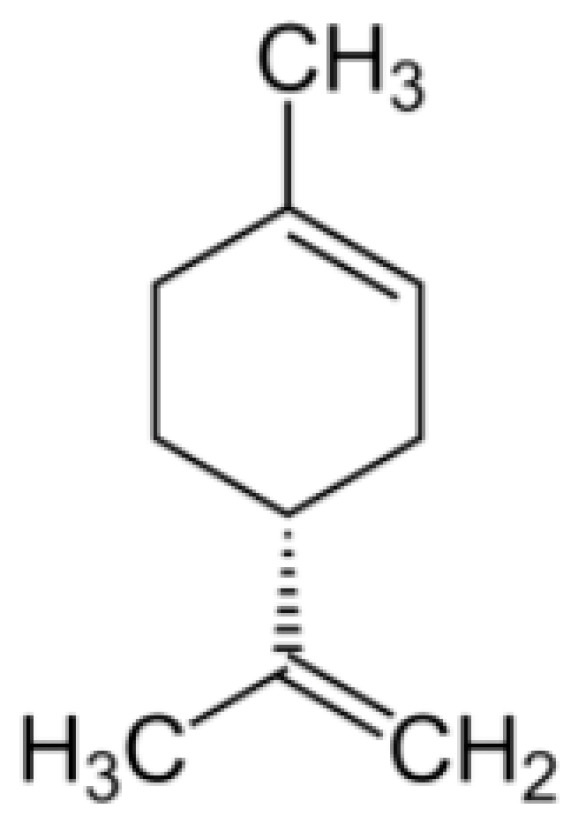
d-Limonene.

d-limonene ((4*R*)-1-methyl-4-isopropenylcyclohex-1-ene) (Figure 6) is one of nature’s most common terpenes, an important constituent in various citrus oils (orange, lemon, mandarin, lime and grapefruit). It can decrease oxidative stress and inflammation [73], has potentially beneficial effects on colon cancer [74], antibacterial activity [75], and neuroprotective properties [76] described in the literature.

d-limonene was administered orally to rats at a dose of 10 mg/kg, and reduced intestinal inflammatory scores and serum TNF-α concentrations compared to rats with untreated TNBS-induced colitis. The anti-inflammatory effect of d-limonene involved the inhibition of TNFα-induced NF-κB translocation in fibroblast cultures. Moreover, when applied to HT-29/B6 colonic cell monolayers, d-limonene increased epithelial resistance [77]. It was seen that d-limonene presents significant anti-inflammatory effects in vivo and in vitro, and its effects involve protection in the epithelial barrier and reduction of cytokines, suggesting a beneficial role of d-limonene in reducing inflammation [77].

In another study by Yu et al. (2017), d-limonene was administered at a dose of 50 or 100 mg/kg p.o. for seven days, and the administration of 2% DSS induced colitis for seven days. Treatment with d-limonene reduced disease activity and colonic mucosa damage. This effect was mediated by suppression of the expression of the matrix metalloproteinase gene (MMP) 2 and 9, zinc-dependent endopeptidases produced by fibroblasts, keratinocytes and inflammatory cells [78]. This reduced levels of inducible nitric synthase expression (iNOS), considered responsible for significantly increasing the production of NO in the epithelium and foci of inflammation in association with nitrotyrosine in UC [79] and COX-2 in rats. Furthermore, it increased the levels of expression of antioxidant proteins and reduced the production of PGE2 and the levels of expression of TGF-β [78].

The administration of d-limonene promoted an increase in the expression levels of kinase regulated by extracellular phosphorylated signal (ERK) 1/2, an important member of the mitogen-activated protein kinase (MAPK) system. The MAPK system plays an essential role in the mediation of inflammatory responses and the regulation of the production of inflammatory cytokines, proliferation and differentiation of epithelial cells, and the inhibition of apoptosis in the intestinal epithelium, indicating its potential antioxidant and anti-inflammatory properties [78].

### 2.7. Menthol

**Figure 7:**
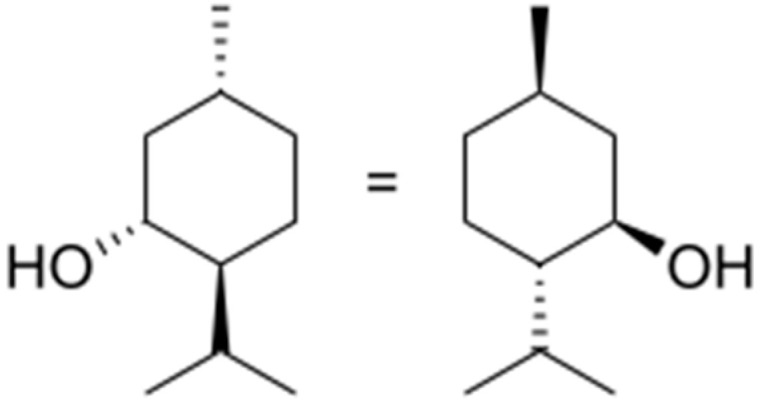
Menthol.

Menthol ((1*R*,2*S*,5*R*)-2-isopropyl-5-methylcyclohexanol) (Figure 7) is a cyclic monoterpene found as the major constituent in the essential oils of *Mentha canadensis* L. (mint) and *M. x piperita* L. (peppermint) [80]. It has pharmacological activities known as an analgesic [81], an effect on bladder hyperactivity and cystitis [82] and as an antioxidant [83].

In the study by Ghasemi-Pirbaluti et al. (2017), the 3% acetic acid induction model was used in rats. This model mainly focuses on oxidative stress in the pathogenesis of colitis, and the administration of the inducing agent generates an imbalance between oxidizing and antioxidant substances [84,85].

Menthol was administered at doses of 20, 50 or 80 mg/kg (p.o.) once daily for three days, starting 24 h before and continuing for two days after the instillation of acetic acid. Additionally, the activity of MPO and cytokines of the inflammatory profile were assessed [86]. In this study, the administration of menthol improved colitis in the acute phase, as indicated by macroscopy, improved histopathological changes, significantly reduced the levels of IL-1β, IL6, and TNF-α, and decreased MPO activity in the colon [86].

### 2.8. Nerol

**Figure 8:**
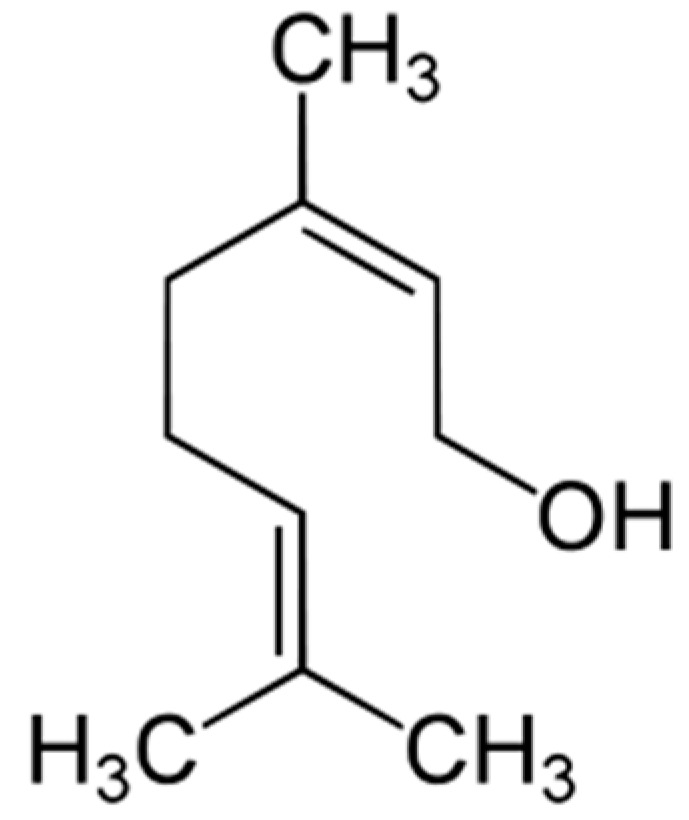
Nerol.

Nerol ((*Z*)-3,7-dimethyl-2,6-octadien-1-ol) (Figure 8) is a natural monoterpene *cis*-isomer of geraniol and the major component of essential oils from spices such as neroli (*Citrus aurantium*), rose (*Rosa damascena*), and lavender (*Lavandula dentata*, *Lavandula stoechas*, and *Lavandula multifida*) [87] with antinociceptive and anti-inflammatory properties [88], as well as antifungal ones [89].

Nerol was administered orally at doses of 10 to 300 mg/kg. The experimental model used in this study was oxazolone induction in mice. This induction represents a model of sepsis and can resemble a severe type of UC, associated with early and severe mucosal lesions and a high mortality rate [90]. The variables measured in animals with oxazolone-induced colitis include weight loss, stool consistency, macroscopic damage and levels of inflammatory cytokines [91].

Nerol (30 to 300 mg/kg) prevented or decreased the pathological changes observed in the colitis model. It also showed antinociceptive effects and reduced the increased levels of inflammatory cytokines (IL-13 and TNF-α) [91]. Among the inflammatory cytokines, TNF-α secreted by several populations of immune and stromal cells is considered responsible for amplifying and maintaining chronic inflammation in IBDs [3]; its inhibition has been used as a therapeutic strategy in IBDs [15].

The results provide evidence of a beneficial effect of nerol in colitis, involving tissue protection, antinociception and immune system modulation, suggesting the therapeutic potential of this monoterpene [91].

### 2.9. Oleanolic Acid

**Figure 9:**
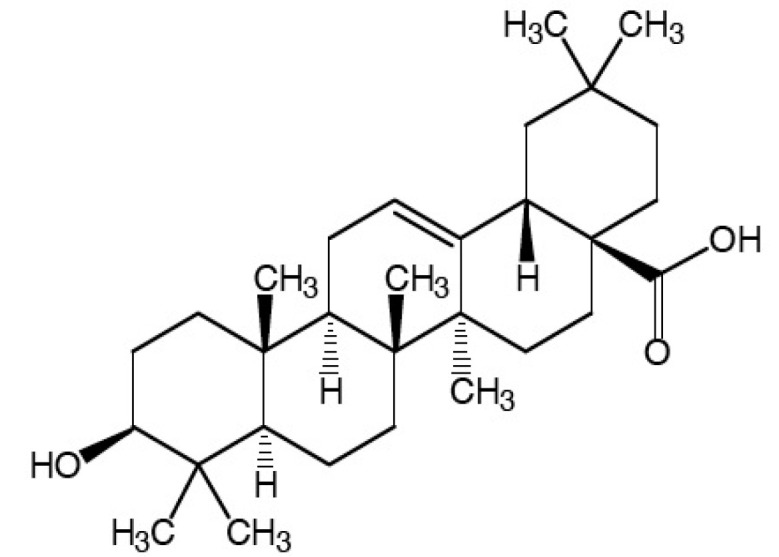
Oleanolic acid.

Oleanolic acid (OA) ((4a*S*,6a*R*,6a*S*,6b*R*,8a*R*,10*S*,12a*R*,14b*S*)-10-hydroxy-2,2,6a,6b,9,9,12um-heptamethyl-1,3,4,5,6,6um,7,8,8-um,10,11,12,13,14b-tetradecahydropicene-4um-carboxylic) (Figure 9).

It is a naturally occurring triterpenoid widely distributed in food and plants of the Oleaceae family, relatively non-toxic, and hepatoprotective [92]. It also has anti-tumor and anti-inflammatory properties [93].

Kang et al. (2015) observed during a screening program for anti-inflammatory agents of natural products that OA potently inhibited the differentiation of splenic T cells into Th17 cells in vitro. From these results, the authors investigated the anti-inflammatory effect of OA in mice in the DSS-induced colitis model.

Oral administration of OA inhibited DSS-induced colon shortening, macroscopic score, and MPO activity. The treatment with OA inhibited the differentiation of cells to Th17 in the colon’s lamina and decreased the expression of RORγt and IL-17 that expressed mainly in cells of the Th17 profile. The differentiation of Treg cells and the expression of Foxp3 and IL-10 were increased by treatment with OA [92].

Treatment with OA increased the expression of occlusion junction proteins, such as ZO-1, ocludine and claudin-1 (KANG et al., 2015), that act in maintaining intestinal integrity [50]. Furthermore, treatment with OA inhibited the expression of TNF-α, IL-1β and IL-17 and the activation of NF-κB and MAPKs. The results suggest that OA can improve the inflammation present in colitis, inhibiting Th17 cell differentiation and increasing Treg cell differentiation [92].

### 2.10. Paeoniflorin

**Figure 10:**
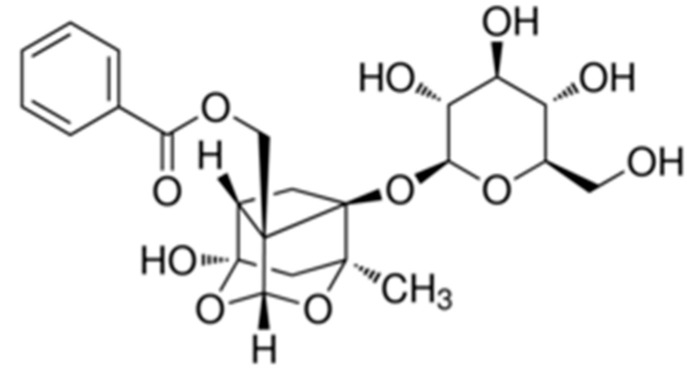
Paeoniflorin.

Paeoniflorin ([(1*R*,2*S*,3*R*,5*R*,6*R*,8*S*)-6-hydroxy-8-methyl-3-[((2*S*,3*R*,4*S*,5*S*,6*R*)-3,4,5-trihydroxy benzoate-6-(hydroxymethyl) oxan-2-yl] oxy-9,10-dioxatetracycle [4.3.1.0 ^2.5^ .0 ^3.8^] decan-2-yl] methyl benzoate) (Figure 10) is a monoterpene glycoside purified from the Chinese herb *Paeonia lactiflora*, with various biological activities described as anti-cancer activity [94], reduced blood viscosity [95] and anti-inflammatory activity [96].

Zhang et al. (2014) evaluated the effect of paeoniflorin on DSS-induced colitis in mice, based on the role that Toll-like receptor 4 (TLR4) plays in intestinal inflammation. They investigated the effect of paeoniflorin on the expression of TLR4 as well as the NF-κB and MAPK.

The induction of paeoniflorin (50 mg/kg, p.o.) reduced the severity of colitis. It resulted in the negative regulation of several inflammatory parameters in the colon, including MPO activity and mRNA expression of mediators of the profile inflammatory such as monocyte chemoattractant protein-1 (MCP-1), COX-2, IFN-γ, TNF-α, IL-6, and IL-17. Experiments using knockdown animals and overexpression of TLR4 demonstrated that TLR4 is a requirement for the negative regulation of inflammatory cytokines [97].

The inhibition of NF-κB p65, ERK, JNK and p38 MAPK activation correlated with a decrease in the TLR4 receptor mucosa, but not in the expression of TLR2 or TLR5. According to the in vivo results, paeoniflorin reduced the expression of TLR4, blocked the nuclear translocation of NF-κB p65, and reduced the production of IL-6 in RAW264.7 cells from macrophages stimulated by LPS [97].

Wu et al. (2019) evaluated the activity of paeoniflorin in an in vitro assay (human Caco-2 cells stimulated by LPS). They showed that paeoniflorin reduces the expression of COX-2, iNOS, TNF-α, IL-6 and MMP-9.

Furthermore, paeoniflorin improved oxidative stress by negatively regulating the NF-κB pathway and by activating the nuclear factor 2 heme oxygenase-1 (Nrf2/HO-1) signaling pathways in Caco-2 cells stimulated by LPS (Wu et al., 2019). The results suggest that paeoniflorin inhibits endothelial damage, has an anti-inflammatory effect, and can be a potential therapeutic agent against IBDs [98].

### 2.11. Perillaldehyde

**Figure 11:**
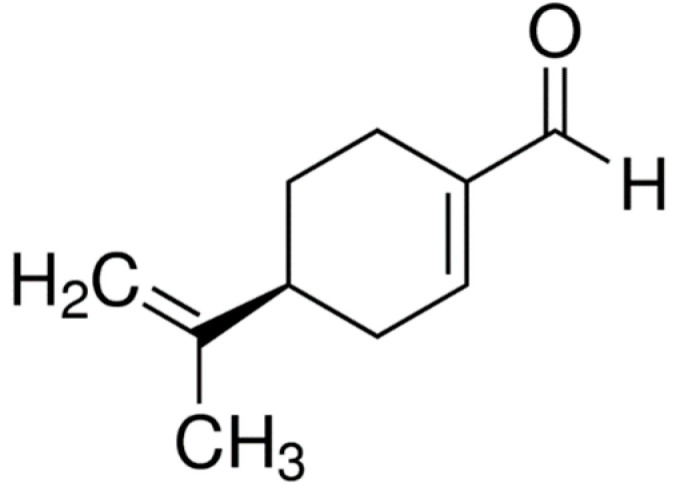
Perillaldehyde.

Perillaldehyde ((*S*)-4-(1-methylethyl)-1-cyclohexene-1-carboxaldehyde) (Figure 11) is a volatile monoterpene, the main component of the essential oil of *Perilla frutescens* leaves. It has biological activities such as vasodilator [99], anti-inflammatory and antidepressant [100], and antifungal [101].

In a study conducted by Uemura et al. (2018), perillaldehyde was administered in doses of 50, 100 or 200 mg/kg orally once daily for up to 17 days, and colitis was induced by 2% DSS solutions. The results showed that the administration of perillaldehyde (100 mg/kg) attenuated the weight loss and the colon damage of the mice.

Furthermore, the administration of this monoterpene resulted in a suppression of the expression of pro-inflammatory cytokine genes (60.6% reduction in TNF-α mRNA levels) and MMP-9 of the colon matrix, as well as suppression of gene expressions and proteins induced by LPS, pro-inflammatory cytokines and c-Jun N-Terminal Kinases (JNKs, p54 and p46) belonging to the MAPK family, whose activation is related to inflammation and cell stress. Thus, perillaldehyde acts to improve intestinal inflammation via JNK-mediated cytokine regulation [102].

### 2.12. Thymol

**Figure 12:**
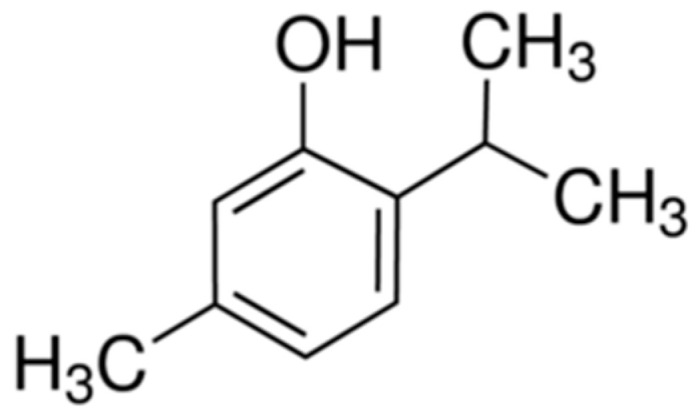
Thymol.

Thymol (5-methyl-2-propan-2-ylphenol) (Figure 12) is a monoterpene found in certain plant species such as thyme [103]. It has proven pharmacological activities such as antioxidant [104], anti-inflammatory [105], analgesic [106], antibacterial [107], antifungal [108], and antitumor [109].

Colitis was induced by intrarectal administration of 2 mL of diluted acetic acid solution (4%) in rats. Colitis was induced on the first day, and treatments started 2 h after colitis induction and continued for five days. Thymol was administered at doses of 10, 30 or 100 mg/kg p.o. per day. Macroscopic and histopathological investigations were performed [110].

Thymol treatment reduced macroscopic and histological damage compared to the control group and markedly inhibited the production of MPO and TNF-α in the colon tissue. Moreover, thymol reduced the expression of the NF-κB p65 protein. The results suggest that thymol exerts an anti-inflammatory effect on colitis, inhibiting the NF-κB signaling pathway and decreasing the expression of TNF-α and MPO activity [110].

In another study by Tahmasebi et al. (2019), luminal instillation of acetic acid was used to induce colitis in rats, and thymol (100 mg/kg p.o.) was administered for ten consecutive days. The expression of COX-2 was evaluated by immunohistochemistry, levels of total protein, NO, MPO, malondialdehyde (MDA), IL-1, IL-6, and TNF-α and relative expression of IκBα and RNAm NF-κBp65 using reverse transcriptase PCR (RT-PCR) of colon homogenates.

COX-2 expression was reduced in animals treated with thymol, with a reduction in MPO activity and MDA levels. The total protein content of the intestine in animals treated with thymol as well as the levels of IL-6 and IL-1 were reduced compared to the control group, and the levels of NF-κBp65 mRNA were also reduced. The results obtained show that thymol can be a promising agent to improve UC in the evaluated model [111].

## 3. Materials and Methods

In this article, we reviewed studies published between January 2010 and April 2020 on terpenes active in IBDs. The research was carried out in databases such as Science Direct^®^ and PubMed^®^, using keywords such as intestinal anti-inflammatory activity or ulcerative colitis or inflammatory bowel diseases and terpenes. The terpenes mentioned in this review were selected according to the pharmacological action demonstrated in specific experimental models to evaluate the intestinal anti-inflammatory activity in animal models or in in vitro studies or studies to elucidate their mechanism of action. We included studies conducted with isolated terpenes and excluded those in which terpenes were components from extracts or fractions to avoid the possible effect of interactions such as synergism or antagonism between compounds.

## 4. Conclusions

**Figure 13:**
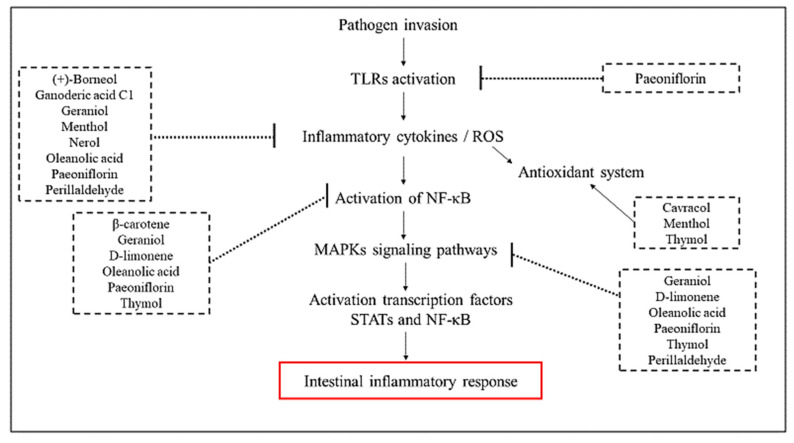
Diagram summarizing molecular mechanisms and pathways where terpenes act as anti-inflammatory agents.

This review showed the intestinal anti-inflammatory profile of terpenes in experimental models related to inflammatory bowel diseases. The reported data suggest the therapeutic potential of natural products, especially of this chemical class, as a source for the development of new therapeutic agents, due to the broad and differentiated mechanisms of action of these metabolites (Figure 13). Considering that the terpenoid compounds discussed in this review are found in many aromatic and medicinal plants, proof of their pharmacological activity may benefit the general population, increasing access to new therapies.
